# Mid-term outcomes after total hip arthroplasty in 106 Crowe II/III hips: different hip center positions

**DOI:** 10.1186/s40001-022-00936-0

**Published:** 2022-12-09

**Authors:** Cheng-Qi Jia, Hong-Fa Pan, Yu-Jie Wu, Yong-Gang You, Shi-Qi Cao, Xue-Song Zhang

**Affiliations:** 1 Medical School of Chinese PLA, 28 Fuxing Road, Haidian District, Beijing, 100853 China; 2 Department of Orthopedics, Chinese PLA General Hospital, 28 Fuxing Road, Haidian District, Beijing, 100853 China; 3 Department of Orthopedics, Weifang People’s Hospital, Shandong, China; 4 Department of Nursing, The Third People’s Hospital of Datong, Shanxi, China; 5 Department of Orthopedics of TCM Clinical Unit, 6Th Medical Center, Chinese PLA General Hospital, Beijing, China

**Keywords:** Surgical technique, Crowe II, Crowe III, Hip center position, Total hip arthroplasty

## Abstract

**Background:**

Under the obvious acetabular superolateral bone defect of Crowe II/III hips, this study aimed to investigate the difference in surgical technique of different hip center positions from the surgical data and clinical outcomes.

**Methods:**

From July 2007 to December 2016, 87 patients (106 Crowe II/III hips) consecutively received total hip arthroplasty (THA). The minimum follow-up time was 5 years. The mean limb length discrepancy was 1.97 ± 1.81 cm. Twenty-four hips had surgical histories. The patients were divided into three groups according to the acetabular prosthesis positions, depending on the Crowe classification, respectively, group 1 (Crowe I), group 2 (Crowe II) and group 3 (Crowe III). The surgical data and clinical results were used to evaluate the outcome of different surgical techniques of different hip center positions, including surgical time, blood loss, blood transfusion, number of osteotomy hips, osteotomy length, the distribution of prothesis, postoperative inpatient days, Harris hip scores, Visual Analogue Scale (VAS), Back Pain Function Scale (BPFS) and complications.

**Results:**

The mean follow-up time was 8.93 ± 2.55 years. Nineteen hips performed intraoperative osteotomy. From group 1 to group 3, the mean osteotomy length were 0.53 ± 1.11 cm, 0.05 ± 0.22 cm, and 0.00 ± 0.00 cm, respectively (*p* = 0.083); the surgical time were 142.57 ± 57.94 min, 118.4 ± 41.22 min, and 120.00 ± 84.85 min, respectively (*p* = 0.324); the blood loss were 498.21 ± 368.53 mL, 333.33 ± 167.62 mL, and 350.00 ± 212.13 mL, respectively (*p* = 0.255); the blood transfusion were 288.48 ± 381.68 mL, 128.00 ± 235.17 mL, and 385.00 ± 219.20 mL, respectively (*p* = 0.199); the postoperative inpatient days were 7.95 ± 4.42 d, 7.47 ± 4.29 d, and 6.50 ± 0.71 d, respectively (*p* = 0.831). Among the groups, the distribution of acetabular prosthesis, acetabular liner, acetabular prosthesis sizes, femoral head sizes and femoral prothesis distal sizes were not significantly different (*p* > 0.05). Only the distribution of femoral prosthesis was significantly different (*p* = 0.046); the Harris, VAS, BPFS, and the distribution of complications were not significantly different (*p* > 0.05).

**Conclusions:**

We provided a framework to guide decision-making in Crowe II/III hips for surgeons: the surgical technique of different hip center positions was stable and had good outcomes, but the acetabular prothesis position and femoral prothesis should be determined according to the intraoperative situation.

**Level of evidence:**

Therapeutic Level II. See Instructions for Authors for a complete description of levels of evidence.

## Introduction

Total hip arthroplasty (THA) is an important operation for patients with developmental dysplasia of the hip (DDH), which not only relieves pain of severe hip osteoarthritis, but also improves hip function for the femoral head dislocation [1]. Currently, though the clinical outcomes of THA in DDH patients have been confirmed [2], corresponding complications like the periprosthetic fractures, limp, knee valgus, knee pain, thigh pain (distal femoral prosthesis), and hip abnormal noise came after THA [3].

An obvious bone defect was frequently found at the superolateral of the true acetabulum in Crowe II/III hips during THA [4]. The special anatomy was like the slab in Rock Climbing, and the slope makes the true acetabulum hard to hold the acetabular cup with host bone coverage in THA. Then, augmentation by structural bone graft to supplement bone insufficiency was commonly required; however, it made the procedure complicated and time consuming. Therefore, the proximal placement of the acetabular component in THA was proposed for enough bone coverage, but influenced the rotational center of the hip, resulting in correlated complications [5]. More recent clinical studies have recommended that the acetabular cup uncoverage should not exceed 30% of its overall surface, otherwise a structural bone grafting may be needed. It was also reported that uncoverage values of less than 24.5% with or without screw was safe for patients with Crowe II/III hips [4]. Even so, different placements of the acetabular prosthesis are still controversial and more mid-term follow-up studies are required.

In the study, structural bone grafting was not perfect, we mainly tried to place the acetabular prosthesis on enough bone contact through surgical technique without structural bone grafting, which was a useful method to hold the acetabular prosthesis [6]. Currently, most research paid more attention to radiograph evaluations or specifically to Crowe IV hips [7], but reliable research with sufficient quantity and mid-term outcomes on surgical technique of different hip center positions in Crowe II/III hips were rare.

Under the obvious acetabular superolateral bone defect of Crowe II/III hips, this study aimed to investigate the difference in surgical technique of different hip center positions from the surgical data and clinical outcomes, rather than postoperative X-rays that are susceptible to measurement.

## Patients and methods

### Study design

From July 2007 to December 2016, 87 Crowe II/III patients (106 hips) consecutively received THA. The inclusion criteria were as follows: 1) THAs for Crowe II/III hips; 2) ≥ 5 year follow-up; and 3) age  > 18 years; and 4) gluteus medius muscle tension was restored during THA. The exclusion criteria were as follows: patients lost to follow-up during 5 years; and systemic rheumatic diseases, ankylosing spondylitis, neurological diseases, psychiatric disorders and other uncontrolled systematic disorders. Institutional review board approval and related informed consent were obtained.

The patients were divided into three groups according to the acetabular prosthesis positions, depending on the Crowe classification [8]: group 1 (Crowe I), group 2 (Crowe II) and group 3 (Crowe III). The surgical data and clinical results were used to evaluate the outcome of different surgical techniques of different hip center positions, including surgical time, blood loss, blood transfusion, number of osteotomy hips, osteotomy length, femoral prosthesis distal size, postoperative inpatient days, Harris hip scores, Visual Analogue Scale (VAS), Back Pain Function Scale (BPFS) [9], and the distribution of acetabular prosthesis, femoral prosthesis, acetabular liner, acetabular prosthesis sizes, femoral prosthesis sizes, hip surgical history, and complications.

Complications at follow-up included: periprosthetic acetabular fractures, periprosthetic femoral fractures, limp, knee valgus, knee pain, thigh pain (distal femoral prosthesis), and hip abnormal noise. Data results were cross-checked by the other two independent orthopedic surgeons.

### Operation procedures

The acetabular prosthesis included Link Betacup (Link, Hamburg, Germany), Link Combicup, Depuy Pinnacle (DePuy, Warsaw, USA), and Depuy Duraloc. The femoral prosthesis included Link LCU, Depuy Corail, Depuy S-rom. The acetabular liner included metal on highly cross-linked polyethylene (MOP), third generation ceramic on ceramic (3rd-COC) and fourth generation ceramic on ceramic (4th-COC). The surgeries were performed by the posterolateral approach. After we resected the femoral head and eliminated fibrous tissue and osteophytes to reveal the true acetabulum, the acetabulum was reamed gradually to achieve the medial wall of the true acetabulum with bleeding spongy bone [10]. Porous-coated acetabular prostheses were placed in the true anatomic acetabular position or higher position with as much host bone coverage as possible. If it was difficult to reset the hip during the surgery, the shortening subtrochanteric osteotomy (SSTO) was performed in case of neurovascular damage [11]. The osteotomy length equaled the distance between the true acetabular center and the femoral head center during the trial reduction minus 15 mm. At the same time, the gluteal muscles must remain adequate tension.

### Statistical analysis

SPSS 26.0 (SPSS Inc) was used for statistical analysis by an independent orthopedic surgeon. Significance was indicated by an α value of  < 0.05. Categorical variables were presented as frequencies and continuous variables as means and standard deviation. Student-Newman–Keuls were performed in continuous variables. Chi-square test or Fisher exact test were performed to determine the difference in categorical variables.

## Results

There were 9 males and 78 females in the study. The mean age was 47.66 ± 12.40 (24–72) years. All patients were followed up  ≥ 5 years (range, 5–14 years). The mean body mass index (BMI) was 62.23 ± 10.01 kg/m^2^. The mean limb length discrepancy was 1.97 ± 1.81 cm (Table 1). There were 24 hips that had hip surgical histories, twenty in group 1, two in group 2, and two in group 3 (*p* = 0.204) (Table 2).

**Table 1:** Patient demographic parameters

Parameters	Crowe II/III (N = 87, 106 hips)
Gender (no. [%])	
Male	9 (10 hips)
Female	78 (96 hips)
Age^†^ (yr)	47.66 ± 12.40 (24–72)
Height^†^(cm)	159.27 ± 7.90 (135–182)
Weight^†^ (kg)	62.23 ± 10.01 (32–96)
BMI^†^ (kg/m^2^)	24.62 ± 4.19 (16–38)
Limb length discrepancy^†^ (cm)	1.97 ± 1.81 (0–10)
Follow-up^†^ (yr)	8.93 ± 2.55 (5–14)

*BMI* Body mass index

^†^The values are given as the mean and the standard deviation

**Table 2:** Surgical data after THA in Crowe II/III Hips

Clinical Factor	Acetabular prosthesis position			*p*
	Crowe I (80 hips)	Crowe II (21 hips)	Crowe III (5 hips)	
Hip surgical history (no. [%])	20	2	2	0.204
Postoperative inpatient days† (d)	7.95 ± 4.42	7.47 ± 4.29	6.50 ± .71	0.831
Surgical time† (minutes)	142.57 ± 57.94	118.4 ± 41.22	120.00 ± 84.85	0.324
Blood loss† (mL)	498.21 ± 368.53	333.33 ± 167.62	350.00 ± 212.13	0.255
Blood transfusion† (mL)	288.48 ± 381.68	128.00 ± 235.17	385.00 ± 219.20	0.199
Osteotomy (no. [%])	18	1	0	0.095
Osteotomy length† (cm)	0.53 ± 1.11	0.05 ± .22	0.00 ± .00	0.083
Acetabular prosthesis (no. [%])				0.357
Duraloc	15	6	2	
Betacup	17	1	0	
Combicup	32	7	2	
Pinnacle	16	7	1	
Femoral prosthesis (no. [%])				0.046*
Link LCU	36	5	0	
Depuy S-Rom	41	13	5	
Depuy corail	3	3	0	
Acetabular prosthesis size (no. [%])				0.672
40 mm	1	1	0	
42 mm	0	1	0	
44 mm	12	4	1	
46 mm	15	2	3	
48 mm	15	4	1	
50 mm	20	4	0	
52 mm	9	3	0	
54 mm	4	1	0	
56 mm	3	0	0	
58 mm	1	1	0	
Femoral head size (no. [%])				0.058
22 mm	1	3	0	
28 mm	24	4	2	
32 mm	23	6	3	
36 mm	32	8	0	
Femoral prosthesis distal size† (mm)	8.46 ± 2.33	9.07 ± 1.96	9.40 ± 1.52	0.239
Acetabular liner (no. [%])				0.348
MOP	3	3	0	
3rd-COC	7	2	1	
4th-COC	70	16	4	

*MOP* Metal on highly cross-linked polyethylene, *3rd-COC* the third generation ceramic on ceramic, *4th-COC* the fourth generation ceramic on ceramic

*p < *0.05*

^†^The values are given as the mean and the standard deviation.

Nineteen hips performed intraoperative osteotomy, 18 in group 1, 1 in group 2 and 0 in group 3 (*p* = 0.095). From group 1 to group 3, the mean osteotomy length were 0.53 ± 1.11 cm, 0.05 ± 0.22 cm, and 0.00 ± 0.00 cm, respectively (*p* = 0.083); the surgical time were 142.57 ± 57.94 min, 118.4 ± 41.22 min, 120.00 ± 84.85 min, respectively (*p* = 0.324); the blood loss were 498.21 ± 368.53 mL, 333.33 ± 167.62 mL, and 350.00 ± 212.13 mL, respectively (p = 0.255); the blood transfusion were 288.48 ± 381.68 mL, 128.00 ± 235.17 mL, and 385.00 ± 219.20 mL, respectively (*p* = 0.199); the postoperative inpatient days were 7.95 ± 4.42 d, 7.47 ± 4.29 d, and 6.50 ± 0.71 d, respectively (*p* = 0.831) (Table 2).

The distribution of acetabular prosthesis, acetabular liner, acetabular prosthesis sizes, femoral head size and femoral prothesis distal size were not significantly different (*p* > 0.05). The Combicup was the most used acetabular prosthesis (39%, 41/106), and the S-Rom was the most used femoral prothesis (59/106, 56%). The 4th-COC was most considered for Crowe II/III hips (90/106, 85%). The acetabular prosthesis sizes were from 40 to 58 mm, 50 mm was the most used (24/106, 23%). And femoral head sizes were from 22 to 36 mm, 36 mm was the most used (40/106, 38%). the femoral prothesis distal size were 8.46 ± 2.33 mm, 9.07 ± 1.96 mm, and 9.40 ± 1.52 mm (*p* = 0.239); Only the distribution of femoral prosthesis was significantly different (*p* = 0.046) (Table 2).

From group 1 to group 3, the mean postoperative Harris were 94.31 ± 5.43, 93.31 ± 4.37, and 93.00 ± 4.18, respectively (*p* = 0.496); the mean postoperative VAS were 0.08 ± 0.38, 0.00 ± 0.00, and 0.00 ± 0.00, respectively (*p* = 0.608); and the mean postoperative BPFS were 59.35 ± 2.75, 60.00 ± 0.00, and 60.00 ± 0.00, respectively (*p* = 0.738) (Table 3).

**Table 3:** Clinical outcomes at 5 years after THA in Crowe II/III Hips

Clinical factor	Acetabular prosthesis position			*p*
	Crowe I (80 hips)	Crowe II (21 hips)	Crowe III (5 hips)	
Postoperative BPFS†	59.35 ± 2.75	60.00 ± .00	60.00 ± .00	0.738
Postoperative Harris†	94.31 ± 5.43	93.31 ± 4.37	93.00 ± 4.18	0.496
Postoperative VAS†	0.08 ± .38	0.00 ± .00	0.00 ± .00	0.608

*BPFS* back pain function scale, *Harris* Harris hip score, *VAS* visual analogue scale

^†^The values are given as the mean and the standard deviation

There were two hips that underwent revision for periprosthetic fracture, one hip for acetabular fracture and another hip for femur fracture. The incidences of complications were 0.9% in revision for acetabular fractures (1/106), and 0.9% in revision for femoral fractures (1/106). Limp still remained in 30.2% (32/106), knee valgus in 13.2% (14/106), knee pain in 2.8% (3/106), thigh pain (distal femoral prothesis) in 2.8% (3/106), and hip abnormal noise in 2.8% (3/106). However, the distribution of complications from group 1 to group 3 were not significantly different (*p* > 0.05) (Table 4).

**Table 4:** Complications at 5 years after THA in Crowe II/III Hips

Complications (no. [%])	Acetabular prosthesis position			*p*
	Crowe I (80 hips)	Crowe II (21 hips)	Crowe III (5 hips)	
Revision for acetabular fractures	0	1	0	0.130
Revision for femoral fractures	1	0	0	0.849
Limp	22	8	2	0.570
Knee valgus	11	2	1	0.790
Knee pain	2	1	0	0.794
Thigh pain (distal femoral prosthesis)	2	0	1	0.050
Hip abnormal noise	2	1	0	0.794

## Discussion

The obvious bone defect at the superolateral of the true acetabulum in Crowe II/III hips influenced the reconstruction of the acetabulum, which made it difficult to achieve acceptable cup coverage at the anatomical acetabulum [12]. On the basis of contemporary advances in primary THA, several methods have been introduced into surgical technique. Though femoral head structural autograft could be utilized at the superolateral rim to provide additional support, it proposed the instability of acetabular component for autologous bone resorption [12]. And the surgical time was also extended accordingly. Then, the high hip center technique was mainly considered in our hospital. And some excellent results were also reported [13].

But reliable research with sufficient quantity and mid-term outcomes on surgical technique of different hip center positions in Crowe II/III hips were rare. Under the obvious acetabular superolateral bone defect of Crowe II/III hips, this study aimed to investigate the difference in surgical technique of different hip center positions from the surgical data and clinical results, rather than postoperative X-rays that are susceptible to measurement.

There are multiple methods for categorizing hip center location in the literature. Dearborn and Harris arbitrarily chosen 35 mm superior to the interteardrop line as a definition for high hip center reconstruction, but they failed to account for variation in pelvic size [14]. Stans et al. measured superior displacement using the approximate femoral head center as a reference point, which took pelvic size into consideration [5]. However, both of them ignored the nature of gradual changes in hip center, polarizing it into two extremes. Then, we divided acetabular prosthesis positions in Crowe II/III hips into three groups according to the Crowe classification for exploring surgical difference in the progressive trend of hip center [8].

### Operation

The surgical time, blood loss, blood transfusion and postoperative inpatient days among the three groups were not significantly different. Some authors indicated that high hip center technique may take a longer time to place the acetabular prothesis and balance the tension of hip muscles; therefore, more surgical time, blood loss, blood transfusion and postoperative inpatient days were needed. However, under friendly surgical tools and rich experience, surgeons could deal with different hip center positions in Crowe II/III hips as original hip center in the conventional THAs, without more surgical trauma. Our study added to the existing body of literature to place the acetabular prothesis in the proper height according to the host bone in THA [13].

The number of osteotomy hips was not significantly different in three groups, but there were 18 hips in group 1 that underwent the SSTO [15], obviously more than that in other groups. And the mean osteotomy length, surgical time, blood loss, blood transfusion and postoperative inpatient days were also little more than that in other groups, but not significantly. This indicated that friendly surgical tools and rich experience were helpful for surgeons and patients [12], but no more other burden. However, most research on Crowe II/III had few reports on these [5, 12].

### Prothesis

The distribution of acetabular prosthesis, acetabular liner, acetabular prosthesis sizes, femoral head size and femoral prothesis distal size were not significantly different. In aspect of surgical technique, the different hip center positions were mainly associated with acetabular prosthesis sizes. Proper hip center position was considered for host bone coverage and matched acetabular prosthesis size. The Combicup was the most used acetabular prosthesis (39%, 41/106) [16]. Screws utilized in Combicup was a technique for stability of acetabular prosthesis under unclear host bone coverage, and more proper sizes could be chosen in Combicup. The acetabular prosthesis sizes were from 40 to 58 mm, 50 mm was the most used (24/106, 23%), which was comparable with conventional THAs. However, no acetabular prosthesis sizes of 38 mm and 60 mm were used in Crowe II/III hips, which was obviously different from that in Crowe IV hips. And femoral head sizes were from 22 to 36 mm, 36 mm was the most used (40/106, 38%), which were more associated with the acetabular prosthesis sizes. S-Rom was the most used femoral prothesis (59/106, 56%) and only the distribution of femoral prosthesis was significantly different. This proved that the special anatomy of straight and narrow femoral canal with an excessive anteversion in Crowe II/III hips should not be ignored, which was really different from conventional THAs [5]. The 4th-COC was most considered for Crowe II/III hips (90/106, 85%). The mean age was 47.66 ± 12.40 years, and the minimum age was 24 years. The wear resistance of acetabular Liner was always a key factor that we must consider for young patients. Though the volumetric wear rates of polyethylene lining were very low 0.022 mm/year, proving highly crosslinked polyethylene was durable in THAs [17] and hip abnormal noise was mainly appeared in ceramic-on-ceramic THAs [18], the advantages of 4th-COC were also obvious in our experience [19]. However, most research on Crowe II/III had few such detailed surgical date [14].

### Function

The postoperative scores of Harris, VAS, and BPFS were not significantly different among three groups, which were different from our original estimate. In aspect of anatomy, placing the hip center in the Crowe I was closest to the normal anatomical structure of the hip, and it should achieve the highest functional scores among three groups. In fact, only Harris in group 1 was only a little higher than that in other groups, but the scores of the VAS and BPFS were a little lower than that in other groups. Then, we can observe that THA not only improved the functional scores in Crowe II/III hips, but also helped relieve the pain from the hip and low back [9]. Besides, the hip center position was not associated with the clinical outcomes in Crowe II/III hips, we should consider more of the host bone coverage on the acetabular prosthesis and actual situation during THA [4]. Our study was consistent with previous research [12]

### Complications

The number of hip surgical history was not significantly different in three groups, so were the complications after THA. The incidences of complications were 0.9% in revision for acetabular fractures (1/106), and 0.9% in revision for femoral fractures (1/106), comparable with 0.4–3.5% in conventional THAs, but younger in age [20]. Different locations of hip center influenced the host bone on the acetabular prosthesis, which may be related to the acetabular fracture. In addition, an over-sized femoral stem in the femoral canal, fractures more easily happened in the event of trauma [21]. However, no aseptic loosening was found in our study, which was consistent with previous reports [5]. In addition, aseptic loosening also occurred in superolateral placement of the cup in some studies [14]. To avoid this situation, the acetabular component was placed medially adjacent to medial wall during THA in our study.

Limp still remained in 30.2% (32/106), but its distribution was not associated with the different hip center positions. Some articles reported that preoperative long-term limb length discrepancy, abductor muscle weakness, and abnormal structure of gluteus medius generally result in limp, and higher acetabular prosthesis position may aggravate them in DDH patients [22]. However, the height of hip dislocation in Crowe II/III hips was not as high as in Crowe IV, these reasons were not confirmed in our study. The number of the limp in group 1 was higher than that in other groups. Some authors indicated that there is a negative correlation of abductor strength with a high rotation center of the hip, for that elevated hip center resulted in a decrease in the muscle length and a corresponding decrease in the preload, leading to weakness of abductor strength [12]. However, in a recent study, restoration of optimal femoral offset and abductor lever arm produced satisfactory results even for a center of hip rotation of  > 30 mm [12].

The knee valgus remained in 13.2% (14/106), which often appeared in DDH patients [11]. When we place the hip center from high dislocation to original anatomy hip center, the medialization of the hip also led to medialization of the knee. The patient would try to walk with the knees closer together to keep the joint line horizontal, then knee valgus appeared. However, knee valgus distribution was not associated with the different hip center positions in our study [23]. Only the number of knee valgus in group 1 was higher than that in other groups.

The knee pain was left in 2.8% (3/106). It often appeared in the early postoperative period and improved with time after THA. It was related to irreversible knee injury from unbalanced walking caused by the unequal length of the lower limbs before surgery. The increased soft tissue tension from restoration of the hip center could aggravate the irreversible knee injury [24]. However, knee pain distribution was not associated with the different hip center positions in our study.

The thigh pain (distal femoral prothesis) remained in 2.8% (3/106), which was lower than that in primary THAs 9–16% [25]. We observed that the high position of the acetabular prosthesis was often for the difficult reduction of the femoral head caused by the high tension of the soft tissue around the hip. Then, strong soft tissue tension could produce the thigh muscle belly pain (distal femoral prosthesis). However, thigh pain distribution was not associated with the different hip center positions in our study. Stem malalignment and the distal contact between the stem tip and the medial femoral cortex also might cause thigh pain [6]. However, S-Rom was the most used in 3 groups 55.7% (59/106), which was more friendly to the femur [12].

The hip abnormal noise was left in 2.8% (3/106), which was comparable with the primary THAs 3.5–36% [18]. Hip abnormal noise mainly appeared in ceramic-on-ceramic THAs in our research, as found in other studies [26]. However, its distribution was not associated with the different hip center positions in our study. This may for that Depuy S-rom was more friendly to the femur and could be easily used to adjust the structural deviation caused by the developmental deformity of the proximal femur [27], then the pressure inside the hip joint could be cushioned.

There are several limitations in the study. First, the sample sizes of the group 2 and 3 were relatively small, but comparable with previous studies. Second, the complications in group 2 and 3 were lower than that in group 1, which could be improved under the continuation of the follow-up work. Third, the follow-up time was still short, it was necessary to continue the follow-up work, especially in aspect of the wear of prothesis. Fourth, there was a lack of more detailed information of prothesis, which could be further improved in the following study.

## Conclusions

We provided a framework to guide decision-making in Crowe II/III hips for surgeons: the surgical technique of different hip center positions was stable and had good outcomes, but the acetabular prothesis position and femoral prothesis should be determined according to the intraoperative situation.

## Data Availability

The data sets generated and/or analysed during the current study are not publicly available due [some of the patient’s data regarding individual privacy, and according to the policy of our hospital, the data could not be shared with others without permission], but are available from the corresponding author on reasonable request.

## Abbreviations

DDH: Developmental dysplasia of the hip
THA: Total hip arthroplasty
BMI: Body mass index
VAS: Visual analogue scale
BPFS: Back pain function scale
MOP: Metal on highly cross-linked polyethylene
3rd-COC: The third generation ceramic on ceramic
4th-COC: The fourth generation ceramic on ceramic
SSTO: The shortening subtrochanteric osteotomy
